# Using micro-computed tomography to examine the larynx in cases of suspected strangulation—a comparison of case findings and control images

**DOI:** 10.1007/s00414-019-02194-y

**Published:** 2019-11-12

**Authors:** Waltraud Baier, Brian A. Burnett, Mark Payne, Jason M. Warnett, Mark A. Williams

**Affiliations:** 1 WMG, International Manufacturing Centre, University of Warwick, Coventry, CV4 7AL UK; 2 UHCW NHS Trust, Coventry, CV2 2DX UK; 3 West Midlands Police, B4 6NQ, Birmingham, UK

**Keywords:** Micro-computed tomography, Forensic Imaging, Strangulation, Larynx

## Abstract

The examination of strangulation is one of the most challenging causes of death diagnoses encountered in forensic pathology. The injuries are often subtle and difficult to detect, especially in cases that lack superficial marks. Fractures of the laryngeal skeleton are commonly regarded as evidence of strangulation but these can be too subtle to be detected during autopsy. Micro-CT is a novel imaging technique that achieves a spatial resolution 1 μm or less which lends itself to the examination of small and delicate structures such as the larynx. However, there is little information to date regarding the appearance of the larynx at this scale, thus complicating the interpretation of the micro-CT images. This study therefore uses micro-CT to examine ten larynges from strangulation deaths and to compare them to nineteen samples from donor individuals in order to distinguish between naturally occurring features and actual trauma. It was found that there are several features which mimic damage in the donor group. Using associated case information, initial trends and patterns of different strangulation methods were established.

## Introduction

Strangulation is the third most common homicide method in the UK, although women are even more likely to become a victim due to intimate partner violence [1, 2]. Despite the frequent occurrence of such cases, the diagnosis of strangulation in forensic pathology is still predominantly one of exclusion, in particular if there is a lack of distinctive signs such as petechial bleeding or bruising of the soft tissues of the neck. Fractures of the laryngeal cartilages or the hyoid bone are considered evidence for strangulation but they can have other causes such as sports injuries, motor vehicle accidents or falls [3]. Such fractures tend to be associated with haemorrhages, but absence thereof can make subtle injuries even more difficult to detect at the risk of potentially missing them during autopsy. In some cases, histological examination is conducted to examine fractures identified at postmortem to assess the timing of the injury in relation to time of death [4]. This process is destructive, time consuming and costly. With advances in technology, new ways of dealing with this issue have emerged with many researchers advocating the use of computed tomography (CT) to study laryngeal trauma [5, 6]. Even more recently, initial studies have explored the use of micro-computed tomography (micro-CT) to examine such cases in order to identify micro-fractures which might not be visible on medical CT or postmortem examination [7, 8]. Micro-CT has proven successful in a number of different forensic applications such as toolmark analysis [9–11], gunshot wound analysis [12, 13] and forensic entomology [14]. However, all of the existing literature on using micro-CT for laryngeal trauma analysis is based on casework; no comparative studies exist to date to the authors’ knowledge. This study aims to address this void as it draws on an unprecedented database of micro-CT scans of larynges from strangulation cases as well as from natural deaths. The injuries observed in the former group were compared with those in the latter control group of uninjured larynges from donor individuals in order to assess which features can occur in the normal population. Studies by Tsai et al. [15] and Baier et al. [16] have compared micro-CT to histology, the current gold standard for forensic injury analysis, and found the method to be reliable for injuries in bone. Method validation is becoming an increasing concern in forensic science and while micro-CT had been validated in industrial settings [17–19] where the method as such has been proven to work, it has not been systematically tested for forensic purposes. This study forms an exploratory study to inform future more structured validation studies.

## Materials and methods

### Micro-CT

The overall principles of micro-CT are similar to medical CT as it uses a stack of 2D radiographs to examine the internal structure of a sample, but it allows a spatial resolution, or voxel size, of approximately 40 μm for a sample of the size of a larynx. The source and detector of a typical lab-based micro-CT machine are stationary during the scan with the sample on a rotating stage at the centre. Radiographs are acquired at every degree angle through a full 360° rotation and later reconstructed to form the 3D model of the object [20].

All samples were scanned using a Nikon 225/320 LC micro-CT scanner (Nikon Metrology, Tring, UK) and reconstructed using the system’s proprietary software CTPro. Typical scan parameters were

120 kV, 135 μA, 500-ms exposure, 24-dB gain, no filtration, creating 3141 projections, although some adjustments were made where necessary. The scan images were visually inspected in VG Studio MAX 2.2 (Volume Graphics, Heidelberg, Germany).

Two groups of larynges were examined. The first consisted of ten samples from forensic strangulation cases. Only cases where strangulation had been confirmed through multiple lines of enquiry (witness statements, confessions, CCTV, forensic post-mortem) were considered in this group; no inconclusive cases were included (age range 20–68 years; male 4, female 6). The second group consisted of nineteen control samples from donor individuals with no recorded history of laryngeal trauma (age range 46–94 years; male 12, female 7). Only the thyroid cartilage and the hyoid were considered for this study. Full demographic details are provided in Appendix Tables 1 and 2.

**Fig. 1:**
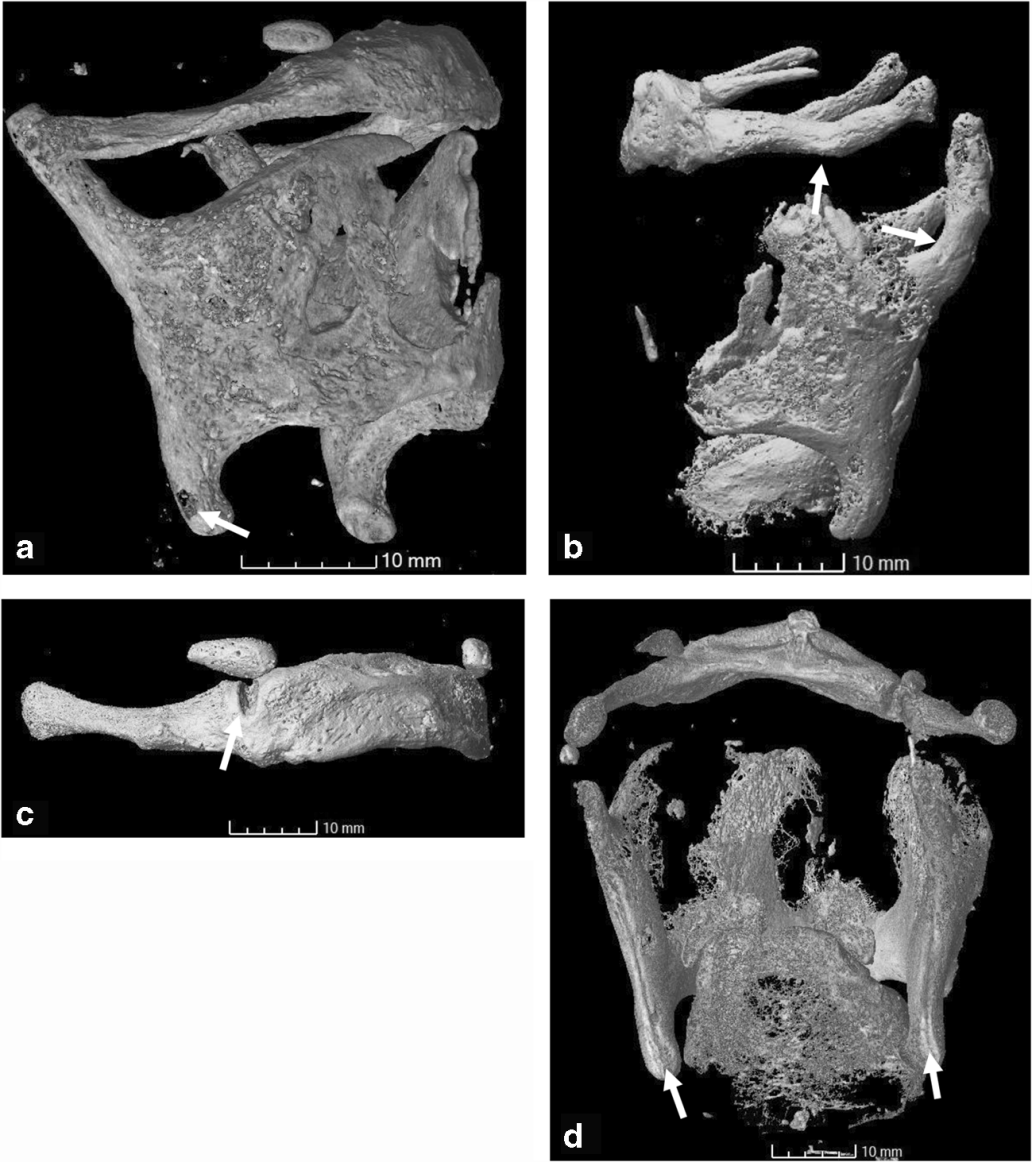
Some of the assessment features described in this study. **a** Circular “hole” within the ossified cartilage and fragmentary ossification, S172809. **b** Unusual angles of the greater horns (hyoid) and superior horns (thyroid cartilage), S173160. **c** Incomplete fusion of the greater horn and body of the hyoid, S170345. **d** Linear crease-like furrow or groove along the posterior margins of the thyroid cartilage, S170452

The examination criteria were determined from the control group as potentially misleading features during analysis. These were circular defects within the ossified cartilage, fragmented appearance of ossified material, incomplete fusion of the hyoid elements, abrupt angles and linear grooves within the ossified cartilage, and the presence of triticeous cartilages. Based on the literature, the presence, completeness (incomplete, complete, displaced) and location of fractures were also included as a criterion as they are frequently encountered in strangulation deaths. In order to include all possible fracture origins, they were termed discontinuities in the analysis. An example for each category is shown in Fig. 1.

The postmortem findings were also noted for the forensic cases. They were summarised as superficial (petechiae, facial congestion, bruising on the neck, ligature marks) and internal (haemorrhage/bruising on the muscles and soft tissues of the neck). The mode of strangulation (manual, ligature, chokehold) was noted where sufficient evidence was available.

## Results

The full results are provided in Appendix Table 1 (strangulation group) and Appendix Table 2 (control group). Triticeous cartilages were observed in 30% of cases in the strangulation group (*n* = 3), and in 47% in the control group (*n* = 9), their appearance being the same in both groups with smooth cortical bone and a spherical to oval shape. Abrupt angles are seen along the superior thyroid margins, the superior thyroid horns, and the greater horns of the hyoid in 10% of cases in the strangulation group (*n* = 1), and 32% of cases in the control group (*n* = 6). Circular holes within the ossified cartilage occurred in 40% of strangulation cases (*n* = 4), and 32% of control cases (*n* = 6), they all display clear and regular margins. Linear grooves were only observed on the posterior aspect (including superior and inferior horns) of the ossified thyroid cartilage (50% or *n* = 5 in strangulation group, 32% or *n* = 6 in control group). They predominantly extend vertically along the posterior margin although some are observed on the lateral aspects of the posterior margin.

**Fig. 2:**
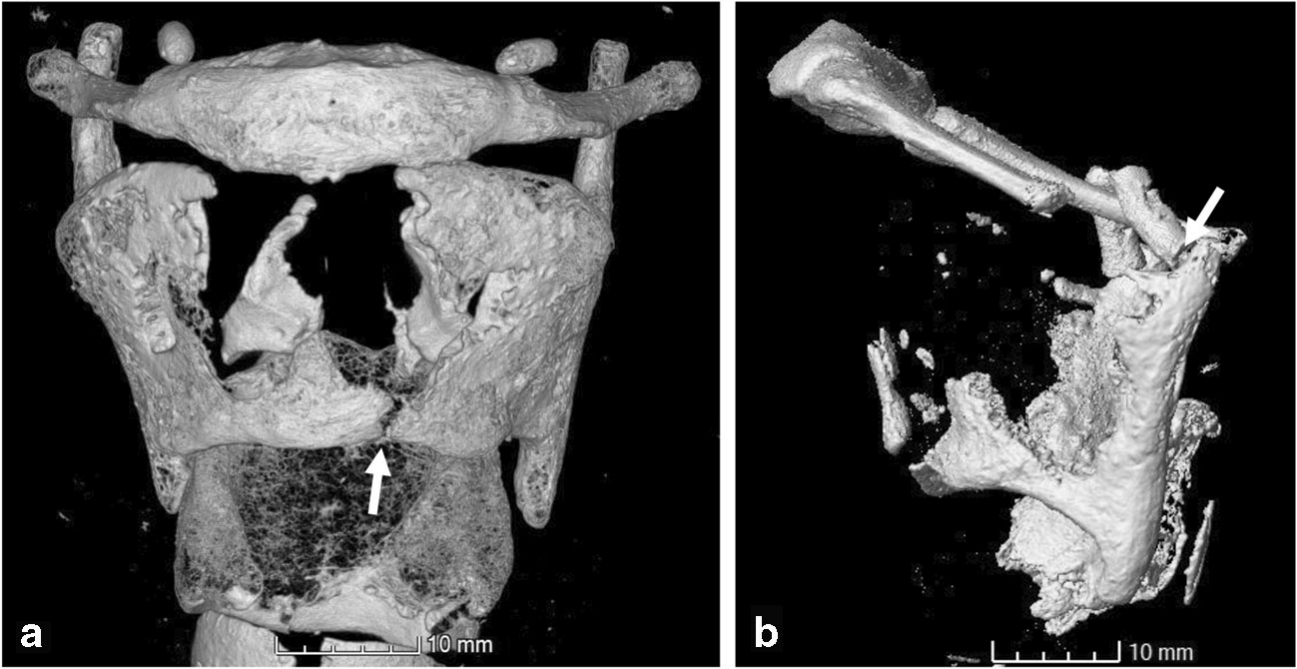
Typical locations of discontinuities identified in this study (arrows). **a** Discontinuity on the inferior thyroid margin of sample S172809 in the control group. **b** Fracture at the base of the superior thyroid horns of the forensic case OP Isengard

Figure 2 illustrates the observation that the forensic cases show discontinuities in three different locations (left and right side were counted as one) whereas there is only one feature location in the control group, on 16% of cases (*n* = 3). Discontinuities at this location were observed in 40% of the forensic samples (*n* = 4), two of which displayed a discontinuity left and right of the midline. Seventy percent of the forensic cases (*n* = 7) displayed a discontinuity on the superior thyroid horns with the base being the most affected area. Of these, six cases displayed bilateral discontinuities (total number of discontinuities *n* = 13). The inferior thyroid horns were only affected in two cases (20%). The three cases (30%) where the hyoid bone was fractured were all caused by ligature strangulation.

## Discussion

### Significance of the results

#### Discontinuities

The most likely explanation for the fracture-like discontinuity on the inferior thyroid margin, observed in both groups, is a product of the natural ossification process. While they appear like fractures on the volume-rendering, on the 2D sections the edges of the discontinuity appear rounded and uninterrupted. All other discontinuities, exclusively encountered in the forensic group, are considered true fractures as they display sharper edges with interrupted cortical bone and trabeculae. Hyoid fractures have only been observed in the posterior third of the greater horn where the bone is delicate and thin. The greater horn has been observed as a common fracture location in several other studies of neck compression [3, 5] and appears to be strong support for compression of the neck. All the fractures of the greater horns of the hyoid occurred in ligature strangulations. This contradicts the review by Dettmeyer et al. [21] who found hyoid fractures to be more common in manual strangulations. The majority of superior thyroid horn fractures occur bilaterally. Only one of those bilateral injuries was not a ligature strangulation. However, there were also cases of ligature strangulation which did not result in bilateral fractures. This is a recurring dilemma in interpreting strangulation deaths that there is much overlap in the injury pattern. Many studies have attempted to classify injuries according to the mode of strangulation [22–24] and while there are some trends there is also overlap which warrants caution not to solely rely on fracture patterns for the interpretation.

#### Circular holes

Smooth-edged circular holes were only seen in the thyroid cartilage but were not restricted to a specific location. Their occurrence within the laminae is a common location for the thyroid foramen which serves as an opening for vessels or nerves [25]. It is possible that the circular openings in other locations fulfil a similar purpose or simply represent ossification irregularities. Their presence in the control group supports an interpretation as a natural cause as opposed to possible fingertip damage.

#### Fragmentation

**Fig. 3:**
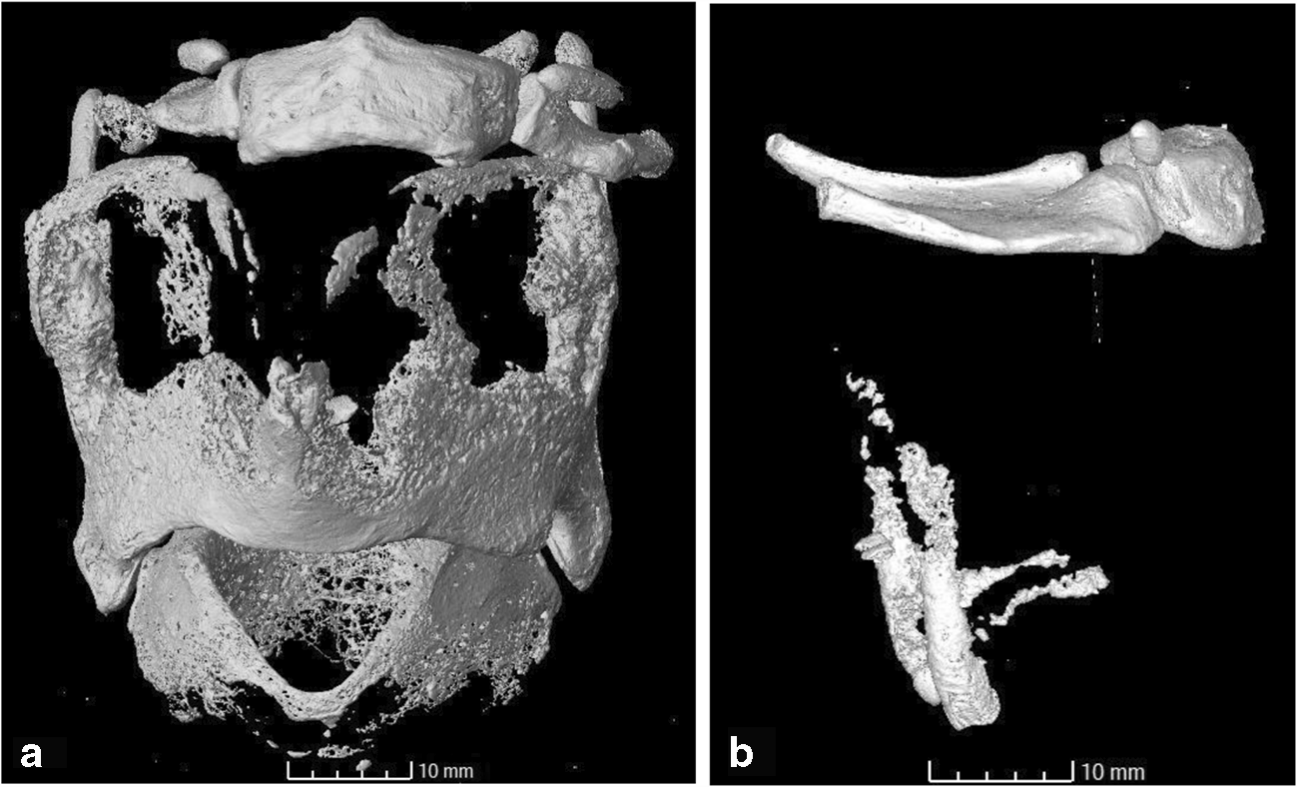
Different stages of laryngeal cartilage ossification. The control group was composed of generally older individuals than the forensic group, displaying more advanced stages of ossification and therefore a different pattern of fragmentation. **a** Sample S170405, showing some fragmentation along the anterior margins of the thyroid laminae. **b** OP Aporia, the individual’s younger age (early 20s) is reflected in the limited ossification and higher degree of fragmentation along the posterior and inferior margins of the thyroid cartilage

The ossified cartilages in the forensic group appeared more fragmented than those in the control group (Fig. 3). This manifested as an increased number of small pieces of ossified material that did not form a coherent structure. However, this could be explained by younger age of individuals in this group. The more advanced age of the donor individuals in the control group is reflected by the more advanced stages of ossification. Nonetheless, the appearance of the loose fragments was similar in both groups. Individual ossified “nodules” displayed rounded edges. While the majority of fragments in the control group are found along the margins of the thyroid laminae, the case examples display fragments in all locations. As they are similar in appearance, they are not considered to be compelling evidence of trauma. No other study has examined this feature at this level of detail to the authors’ knowledge, further comparison will therefore depend on a future expansion of this database.

#### Incomplete fusion

**Fig. 4:**
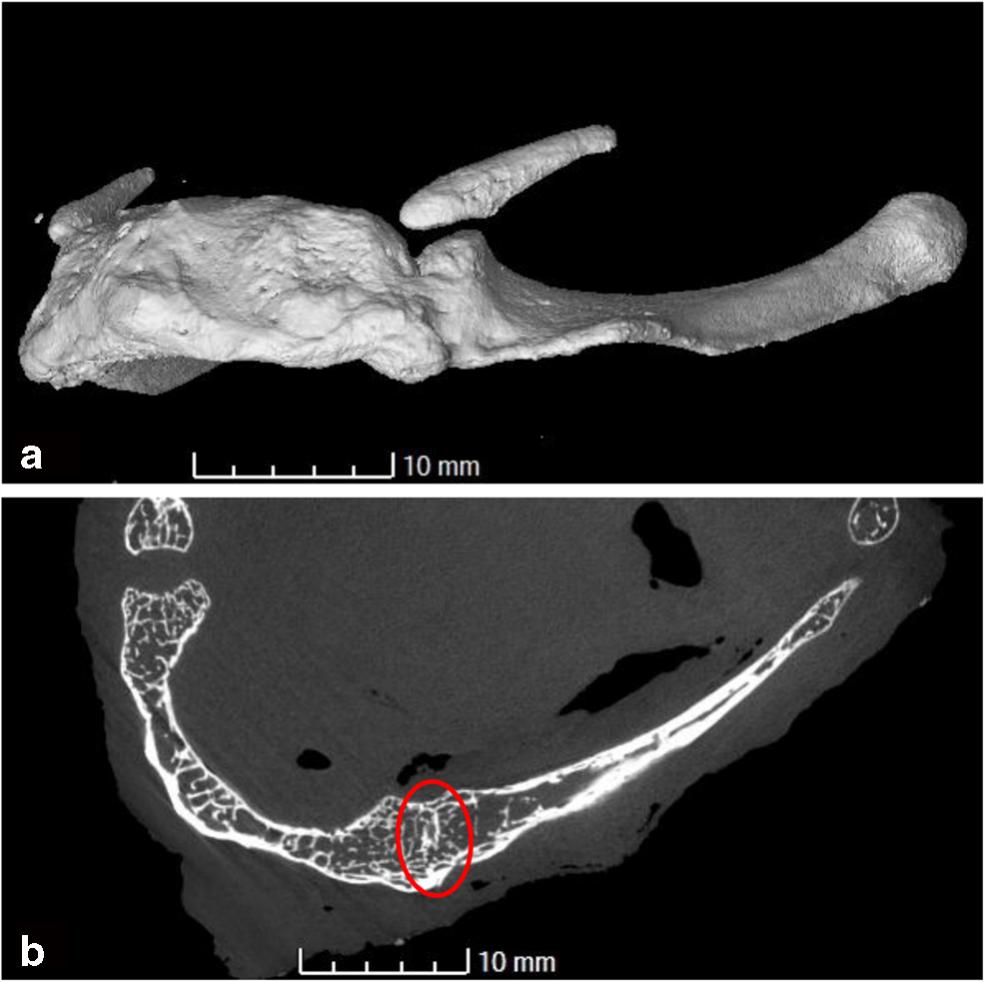
Incomplete fusion of the left greater horn and the body of the hyoid in control sample S171263. **a** Volume rendering showing a bony bridge across the two elements. **b** Transverse section through the specimen at the location of the bridge. The area of fusion still displays some of the original cortical bone surfaces which are gradually being resorbed (circled area)

The fusion or non-fusion of the greater horns to the body of the hyoid bone is an easily misinterpreted feature. While it is well-established in the literature that these elements can fuse over time [26, 27], in pathological practise hypermobility is sometimes taken as evidence of a hyoid fracture [4]. Depending on the pathologist’s sensitivity and experience, a natural non-union could be mistaken for trauma. Radiologically, a narrow gap between the body and greater horn or incomplete fusion of the two parts might be mistaken as a fracture. The control group has shown several examples of incomplete fusion with a narrow gap still visible at the body-greater horn junction, see Fig. 4 for a transverse section and the volume-rendering of the scan. Both sides display an uninterrupted cortical bone surface which only begins to disappear after fusion has completed. No actual fractures were observed at this location in either group.

#### Abrupt angles

These were encountered frequently in the control group, often on the superior thyroid horns and along the superior margins of the thyroid laminae. In one control sample, the greater horns of the hyoid were angled upwards in the posterior third. These angles are present in the normal population but from this study it does not become clear whether they are due to normal variation or previous, healed trauma. A study by Maxeiner [28] found that healed fractures of the hyoid and thyroid cartilage can be found amongst alcoholics who are prone to frequent falls or accidents. However, information about the donors’ lifestyles was not available in the present study.

#### Linear grooves

**Fig. 5:**
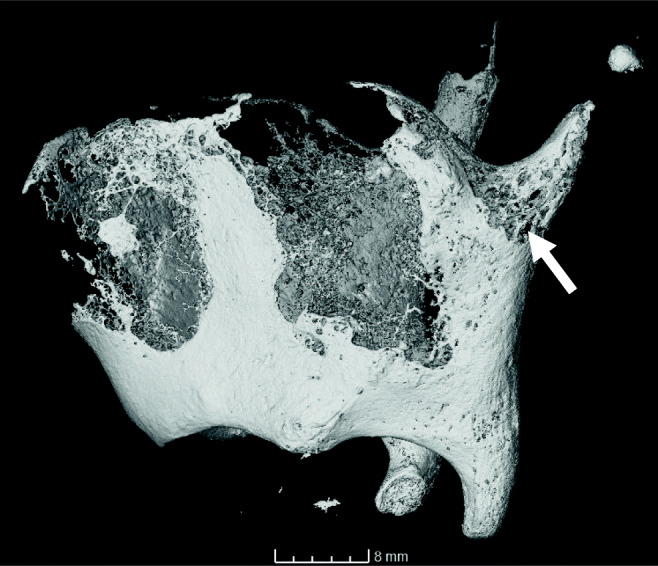
Different ossification within the same specimen (S170452). The area at the base of the superior thyroid horn (arrow) is less densely ossified than the surrounding areas. This contrast might affect the fracture potential at this location

These features resemble fine fissures or creases when seen on the 3D volume-rendered scan, shown in Fig. 2D. Using the 2D sections helped identifying them as superficial. They were frequently observed in the control group which is important to avoid misinterpretation as real fractures in the forensic samples. The location and orientation of these creases differed from the actual fractures observed (vertical as opposed to approximately horizontal). This feature in particular has only been made possible to study through the increased detail of micro-CT and has not been described previously.A similar feature was a sharp crest running in no particular direction, possibly indicating the ossification “front”. One side of this crest displays a cortical bone surface whereas the other side displays less solidly ossified material, shown in Fig. 5 for control sample S170452. When examining the volume rendering of a scan, the shadow created by the crest can mimic a fracture line. Having seen this feature in numerous control cases serves as reassurance that this is a natural feature. This was most commonly observed in the area at the base of the superior thyroid horns. The horns and the posterior margins displayed more densely ossified material compared to the more loosely structured trabeculae at the base of the horns. This contrasting ossification pattern is important to understand as it might impact laryngeal fractures. Saternus et al. [29] argue that the differences in ossification density create weak links which are then more prone to fracturing. A fracture in these areas would therefore not be unexpected and potentially requires less force than in other areas or if ossification there was more homogenous. The base of the superior thyroid horns was frequently seen as less densely ossified in the control group and fractured in the strangulation group, supporting this theory.

#### Triticeous cartilages

These have occasionally been observed in the literature on normal anatomical variation of the larynx [26]. These small spherical ossifications are found in the thyrohyoid membrane which connects the superior thyroid horns to the posterior greater horns of the hyoid. The close proximity to the superior thyroid horns poses the risk of misinterpreting them as fragments of the horns, in particular if the soft tissue structures have not been visualised sufficiently to allow a distinction or if their natural position has been disturbed by the dissection process. The main difference to fragments of the superior thyroid horns is that the triticeous cartilages appear rounded with a smooth cortical surface whereas horn fragments have at least one irregular aspect.

### General discussion

It becomes immediately obvious that the spatial resolution of micro-CT affects the appearance of the ossified laryngeal structures compared to hospital CT. This applies in particular to the volume rendered images, as seen in the illustrations used by Dang-Tran et al. [30] who used medical CT to study the ossification process of the laryngeal cartilages. Micro-CT scans show individual trabeculae and their connective structure while the medical CT scans show these structures only as less well-defined rounded surfaces which has the clear potential to miss subtle fractures. This justifies conducting this study in order to understand this new level of detail and the potential for injury detection the method offers. Studies such as the present one also form an important part of the overall method validation which is elementary to the admission of any new method as evidence before a court of law. In the US, the Daubert standards outline that new methods need to be tested (Daubert v Merrell Dow Pharmaceuticals, Inc 509 U.S.579 (1993)); in the UK, this falls under the guidelines set out by the Forensic Science Regulator [31]. The FSR’s role is to ensure that forensic science practitioners adhere to strict quality standards such as using only validated and verified methods, detailed in the Code of Practise [30]. The FSR does not currently hold statutory powers, but these requirements have also been included in the Criminal Procedure Rules (2015) which are legally binding. They serve to direct courts towards more rigorous scrutiny of forensic science evidence in order to reduce the risk of wrongful convictions to which flawed forensic science has contributed significantly in the past [32]. The present study provides initial image data for both injured and uninjured larynges. This does not yet constitute full validation but rather a prerequisite to design future validation studies as it provides fundamentals of what to expect from such scans. Ultimately, adding new information about minute details of the larynx it aims to provide a more solid knowledge foundation onto which expert witnesses can base their interpretation of strangulation deaths, thus increasing the overall evidence quality. However, due to the normal anatomical variation of the human larynx, more control samples can be sourced to further study these features and establish a rigorous validation process. Such a process would include histological assessment of all the fractures and fracture-mimicking features identified in the control group to verify these. Some of the forensic cases have subsequently been examined histologically as part of the criminal investigation but the features identified on the micro-CT images were not sectioned systematically. Previous research suggests that there is a close match between micro-CT and histology for samples including larynges [16], adding credibility to the present findings. The lack of a systematic comparison with histology could be considered a limitation of the present study. This study has identified many features not previously described in the literature, developing suitable terminology therefore relied solely on the appearance in the micro-CT images.

## Conclusion

This study has demonstrated the importance of studying a control population for comparison when introducing a new method into the forensic examination of strangulation deaths. Some of the features observed on the forensic samples, in particular the discontinuities on the inferior thyroid margin, might be misinterpreted as trauma had they not been seen as naturally occurring in the control group. True fractures were only observed on the posterior aspect of the thyroid cartilage and hyoid bone. Misinterpretation of these features could have dramatic consequences if someone was wrongfully convicted of murder based on such findings, thus making future validation studies based on the present results inevitable.

## Appendix

**Table 1:** Features and their location observed in the suspected strangulation cases along with the autopsy findings

Case	Sex	Age	Feature location	Feature type	Superficial marks	Internal marks	Mode of strangulation
Aponi	F	35	R sup horn	Complete fracture	Yes	Yes	Unknown
			L + R inf thyroid horns	Circular hole			
			Sup thyroid margins	Fragmentary ossification			
			R post thyroid margin	Linear crease			
Albatross	M	32	L sup horn	Displaced fracture	Yes	Yes	Ligature
			R sup horn	Complete fracture			
			R of ant midline inf thyroid margin	Incomplete fracture			
			L of ant midline inf thyroid margin	Incomplete fracture			
			Laminae	Fragmentary ossification			
			L Lamina	Circular hole			
Bulblet	M	71	L greater horn	Complete fracture	Yes	Yes	Ligature
			R sup horn	Incomplete fracture			
			R greater horn	Abrupt angle			
			R	Triticeous cartilage			
Catni	F	61	R of ant midline inf thyroid margin	Complete fracture	Yes	No	Unknown
			L post thyroid margin	Linear crease			
			L base sup thyroid horn	Linear crease			
Chaucer	M	20	L inf horn	Complete fracture	Yes	Yes	Chokehold
			R inf horn	Complete fracture			
			Post thyroid margins	Fragmentary ossification			
Isengard	F	37	L sup horn	Displaced fracture	Yes	Yes	Unknown
			R sup horn	Displaced fracture			
			L + R post thyroid margins	Linear crease			
Marshside	F	51	R greater horn	Complete fracture	Yes	No	Ligature
			R inf horn	Incomplete fracture			
Pencil	M	59	R greater horn	Complete fracture	Yes	Yes	Ligature
			L greater horn	Complete fracture			
			R of ant midline inf thyroid margin	Incomplete fracture			
			R sup horn	Incomplete fracture			
			L sup horn	Incomplete fracture			
			Sup thyroid laminae	Fragmentary ossification			
			L + R	Triticeous cartilages			
			L + R post thyroid margins	Linear crease			
Platte	F	68	L sup horn tip	Displaced fracture	Yes	Yes	Ligature
			R sup horn	Displaced fracture			
			L sup horn base	Complete fracture			
			L of ant midline inf thyroid margin	Complete fracture			
			Laminae	Fragmentary ossification			
			L Lamina	Circular hole			
			R greater horn	Incomplete fusion			
Syringe	F	48	R sup horn	Displaced fracture	Yes	Yes	Manual and ligature
			L sup horn	Displaced fracture			
			R	Triticeous cartilage			
			L + R sup thyroid horns	Fragmentary ossification			
			R inf thyroid horn	Circular hole			
			R lateral post thyroid margin	Linear crease			

**Table 2:** Features and their location observed in the control group, along with demographic details and cause of death as provided by the tissue supplier

Specimen	Sex	Age	Feature location	Feature type	**Cause of death**
S170459	F	81	R of ant midline inf thyroid margin	Complete fracture	Lung cancer
			L + R Lamina	Circular hole	
			Sup thyroid margins	Fragmentary ossification	
			Ant lamina	Fragmentary ossification	
S173045	F	70	R of ant midline inf thyroid margin	Complete fracture	Breast cancer
			Laminae	Fragmentary ossification	
			L lamina	Circular hole	
S172809	F	93	L of ant midline inf thyroid margin	Complete fracture	OP Complications
			R inf thyroid horn	Circular hole	
S170452	M	73	L + R	Triticeous cartilages	Lymphoma
			Sup margins laminae	Fragmentary ossification	
			L + R post thyroid margins	Linear crease	
S170445	M	80	L + R	Triticeous cartilages	Respiratory failure
			L + R post thyroid margins	Linear crease	
S170320	M	75	L + R	Triticeous cartilages	Myeolodysplastic Syndrome
			L post thyroid margin	Linear crease	
S170167	M	90	L + R	Triticeous cartilages	Pulmonary Fibrosis
			Post thyroid margins	Abrupt angles	
			L + R Laminae	Circular holes	
S170163	M	46	Sup margins laminae	Fragmentary ossification	Renal disease
			Sup thyroid horns	Fragmentary ossification	
			L + R	Triticeous cartilages	
S170105	F	86	Ant midline	Fragmentary ossification	Cerebrovascular accident
			L Lamina	Circular hole	
			L + R lateral sup thyroid horns	Linear crease	
S173160	F	75	Sup thyroid horns	Abrupt angles	Neoplasm of the lung
			Greater horns	Abrupt angles	
			Ant and sup laminae	Fragmentary ossification	
S173053	M	79	L sup thyroid horn	Abrupt angle	Lymphoma
S173046	M	88	L sup thyroid horn	Circular hole	Coronary Artery Disease
			Laminae	Fragmentary ossification	
			R sup thyroid margin	Abrupt angle	
S172982	M	88	Sup thyroid margins	Fragmentary ossification	Acute Myocardial Infarction
			Sup thyroid margins	Abrupt angles	
C171288	F	94	Sup thyroid margins	Abrupt angles	Acute Myocardial Infarction
			R greater horn	Incomplete fusion	
			L + R post thyroid margins	Linear crease	
C171258	F	76	Laminae	Fragmentary ossification	Heart failure
			R	Triticeous cartilage	
L172291	M	86	L + R	Triticeous cartilages	Adenocarcinoma of lung
			L post thyroid margin	Linear crease	
S170345	M	86	L + R	Triticeous cartilages	Myeolodysplastic Syndrome
			Laminae	Fragmentary ossification	
S171263	M	67	L greater horn	Incomplete fusion	Leukaemia
S170405	M	87	L + R	Triticeous cartilages	Colon cancer
			Sup margin ant laminae	Fragmentary ossification	

## References

[1] Office for National Statistics (2018) Homicide in England and Wales: year ending March 2017. https://www.ons.gov.uk/peoplepopulationandcommunity/crimeandjustice/articles/homicideinenglandandwales/yearendingmarch2017. Accessed 27 August 2018

[2] Strack GB (2007). How to improve your investigation and prosecution of strangulation deaths.

[3] Dunsby AM, Davison AM (2011). Causes of laryngeal cartilage and hyoid bone fractures found at postmortem. Med Sci Law..

[4] Davison AM, Williams EJ (2012). Microscopic evidence of previous trauma to the hyoid bone in a homicide involving pressure to the neck. Forensic Sci Med Pathol..

[5] Kempter M, Ross S, Spendlove D, Flach P, Preiss U, Thali M, Bolliger S (2009). Post-mortem imaging of laryngohyoid fractures in strangulation incidents: first results. Leg Med..

[6] Becker M, Leuchter I, Platon A, Becker CD, Dulguerov P, Varoquaux A (2014). Imaging of laryngeal trauma. Eur J Radiol..

[7] Kettner M, Potente S, Schulz B, Knauff P, Schmidt PH, Ramsthaler F (2014). Analysis of laryngeal fractures in decomposed bodies using microfocus computed tomography (mfCT). Forensic Sci Med Pathol..

[8] Fais P, Giraudo C, Viero A, Miotto D, Bortolotti F, Tagliaro F, Montisci M, Cecchetto G (2016). Micro computed tomography features of laryngeal fractures in a case of fatal manual strangulation. Leg Med..

[9] Thali MJ, Taubenreuther U, Karolczak M, Braun M, Brueschweiler W, Kalender WA, Dirnhofer R (2003). Forensic microradiology: micro-computed tomography (Micro-CT) and analysis of patterned injuries inside of bone. J Forensic Sci..

[10] Baier W, Norman DG, Warnett JM, Payne M, Harrison NP, Hunt NC, Burnett BA, Williams MA (2017). Novel application of three-dimensional technologies in a case of dismemberment. Forensic Sci Int..

[11] Norman DG, Watson DG, Burnett BA, Fenne P, Williams MA (2017). The cutting edge- micro-CT for quantitative toolmark analysis of sharp force trauma to bone. Forensic Sci Int..

[12] Fais P, Giraudo C, Viero A, Amagliani A, Viel G, Montisci M, Miotto D, Cecchetto G (2015). Identification of bullet entrance in different type of intermediate firearm wounds through micro-computed tomography analysis. J Forensic Radiol Imaging..

[13] Cecchetto G, Giraudo C, Amagliani A, Viel G, Fais P, Cavarzeran F, Feltrin G, Ferrara SD, Montisci M (2011). Estimation of the firing distance through micro-CT analysis of gunshot wounds. Int J Leg Med..

[14] Richards CS, Simonsen TJ, Abel RL, Hall MJR, Schwyn DA, Wicklein M (2012). Virtual forensic entomology: improving estimates of minimum post-mortem interval with 3D micro-computed tomography. Forensic Sci Int..

[15] Tsai A, McDonald AG, Rosenberg AE, Gupta R, Kleinman PK (2014). High-resolution CT with histopathological correlates of the classic metaphyseal lesion of infant abuse. Pediatr Radiol..

[16] Baier W, Mangham C, Warnett JM, Payne M, Painter M, Williams MA (2019). Using histology to validate micro-CT findings of trauma in three post-mortem samples- First steps towards method validation. Forensic Sci Int..

[17] Brunke O, Santillan J and Suppes A (2010) Preecise 3D dimensional metrology using high-resolution x-ray computed tomography (μCT). In: Stock SR (ed) *Proceedings of the SPIE, Volume 7804.* [Online]. SPIE Digital Library. https://www.spie.org/Publications/Proceedings/Volume/7804?&origin_id = x4323&start_yea r = 2010&end_year = 2010&start_at = 41&SSO = 1. Accessed 17 March 2006

[18] Kruth J-P, Bartscher M, Carmignato S, Schmitt R, De Chiffre L, Weckenmann A (2011). Computed tomography for dimensional metrology. CIRP Annals-Manufacturing Technology..

[19] Lifton JJ, Malcolm AA, McBride JW, and Cross KJ (2013) The application of voxel size correction in X-ray computed tomography for dimensional metrology*. 2nd Singapore International Non-destructive Testing Conference and Exhibition*, 19-20 July 2013, Singapore

[20] Welkenhuyzen F, Kiekens K, Pierlet M, Dewulf W, Bleys P, Kruth J-P, Voet A, Dirckx J, Buytaert J (2009). Industrial computer tomography for dimensional measurement: overview of influence factors and improvement strategies. 4th International Conference on Optical Measurement Techniques for Structures and Systems.

[21] Dettmeyer RB, Verhoff MA, Schütz HF (2014). Forensic medicine: fundamentals and perspectives.

[22] Maxeiner H, Bockholdt B (2003). Homicidal and suicidal ligature strangulation- a comparison of the post-mortem findings. Forensic Sci Int..

[23] Naik SK, Patil D (2005). Fracture of hyoid bone in cases of asphyxial deaths resulting from constricting force round the neck. J Indian Academy Forensic Med..

[24] Shaik MM, Chotaliya HJ, Modi AD, Parmar AP, Kalele SD (2013). A study of gross postmortem findings in cases of hanging and ligature strangulation. J Indian Academy Forensic Med..

[25] Raikos A, Paraskevas GK (2013). The thyroid foramen: a systematic review and surgical considerations. Clinical Anat..

[26] Di Nunno N, Lombardo S, Costantinides F, Di Nunno C (2004). Anomalies and alterations of the hyoid-larynx complex in forensic radiographic studies. American J Forensic Med Pathol..

[27] Naimo P, O’Donnell C, Bassed R, Briggs C (2013). The use of computed tomography in determining developmental changes, anomalies, and trauma of the thyroid cartilage. Forensic Sci Med Pathol..

[28] Maxeiner H (1999). Healed fractures of the larynx and lingual bone in forensic autopsy. Archiv fur Kriminologie..

[29] Saternus K-S, Maxeiner H, Kernbach-Wighton G, Koebke J (2013). Traumatology of the superior thyroid horns in suicidal hanging- An injury analysis. Leg Med..

[30] Dang-Tran KD, Dedouit F, Joffre F, Rougé D, Rousseau H, Telmon N (2010). Thyroid cartilage ossification and multislice computed tomography examination: a useful tool for age assessment?. J Forensic Sci..

[31] Regulator FS (2017). Code of practice and conduct.

[32] Cole SA (2012). Forensic science and wrongful convictions: from exposer to contributor to corrector. New Engl Law Review..

